# Sleep Disturbance in Bipolar Disorder: Neuroglia and Circadian Rhythms

**DOI:** 10.3389/fpsyt.2019.00501

**Published:** 2019-07-18

**Authors:** Luca Steardo, Renato de Filippis, Elvira Anna Carbone, Cristina Segura-Garcia, Alexei Verkhratsky, Pasquale De Fazio

**Affiliations:** ^1^Psychiatric Unit, Department of Health Sciences, University Magna Graecia, Catanzaro, Italy; ^2^Department of Medical and Surgical Sciences, University Magna Graecia of Catanzaro, Catanzaro, Italy; ^3^Faculty of Biology, Medicine and Health, The University of Manchester, Manchester, United Kingdom; ^4^Achucarro Center for Neuroscience, IKERBASQUE, Bilbao, Spain

**Keywords:** astroglia, microglia, oligodendroglia, bipolar disorder, depressive behavior

## Abstract

The worldwide prevalence of sleep disorders is approximately 50%, with an even higher occurrence in a psychiatric population. Bipolar disorder (BD) is a severe mental illness characterized by shifts in mood and activity. The BD syndrome also involves heterogeneous symptomatology, including cognitive dysfunctions and impairments of the autonomic nervous system. Sleep abnormalities are frequently associated with BD and are often a good predictor of a mood swing. Preservation of stable sleep–wake cycles is therefore a key to the maintenance of stability in BD, indicating the crucial role of circadian rhythms in this syndrome. The symptom most widespread in BD is insomnia, followed by excessive daytime sleepiness, nightmares, difficulty falling asleep or maintaining sleep, poor sleep quality, sleep talking, sleep walking, and obstructive sleep apnea. Alterations in the structure or duration of sleep are reported in all phases of BD. Understanding the role of neuroglia in BD and in various aspects of sleep is in nascent state. Contributions of the different types of glial cells to BD and sleep abnormalities are discussed in this paper.

## Introduction

Bipolar disorder (BD) is a recurrent disorder that affects in excess of 1% of the world population and usually has its onset in young age. The resulting cognitive deficits, the high risk of suicide, and the occurrence of severe psychiatric and medical comorbidities all make BD one of the major causes of mortality and disability worldwide (1). The concept of BD was introduced at the end of the 19th century by Emil Kraepelin (2) who referred to this disorder as “manic depressive insanity.” About 70 years later, the term “bipolar” was proposed to indicate the condition in which both depression and mania, the opposite poles of mood, alternate in the course of the illness (3). In modern psychiatry, BD is conceptualized as a cyclical mood disorder involving episodes of mania, hypomania, and alternating or intertwining episodes of depression. The last edition of the *Diagnostic and Statistical Manual of Mental Disorders* (DSM-5) categorizes clinical features of BD according to severity (4). Classical BD type I is identified by the occurrence of major depression and full-blown manic episodes, whereas in BD type II, depression is more prominent, with interspersed episodes of less severe manic symptoms, classified as hypomanic episodes. However, despite this general description, the clinical presentation of BD is polymorphic with regard to symptomatology, progression, efficacy of therapies, and functional outcome. Consequently, the DSM-5 introduces additional specifics for diagnosis, such as BD “with mixed features,” or “with rapid cycling,” or “with melancholic features,” or “with mood congruent or incongruent psychotic features,” to mention only a few. Far from being a discrete diagnostic entity, there is increasing recognition of a spectrum of BDs that ranges from marked and severe mood disturbance into milder mood variations (5). In this context, “cyclothymia” is the term assigned to recurrent hypomanic episodes and subclinical episodes of depression. It represents a subsyndromal condition, although mood disturbance is a continuing problem and interferes with everyday functioning (5). Moreover, unlike previous versions that included BD along with all other mood disorders, DSM-5 now assigns a separate chapter to BD and places it between depressive disorders and the spectrum of schizophrenia and other psychotic disorders. The rationale for this new diagnostic taxonomy stems from the assumption that BD could be considered as a bridge that, in terms of genetics, familiarity, and clinical picture, holds together the other two pathologies, sharing some clinical aspects of both.

Despite numerous studies performed in recent decades, little is known about the etiopathogenetic mechanisms responsible for the BD. The most recent research is focusing on the possible biologic mechanisms underlying the disorder, including genetic components, neurochemical abnormalities, and morphostructural brain differences, along with psychosocial factors, such as life experience and social environment context (6). Hitherto, there is no sufficient explanation to account for the pathobiology of such a multiform condition while the disease heterogeneity prompts us to contemplate multifactorial genesis. Indeed, no single paradigm can explain the occurrence and the variability in course and severity of manic-depressive disorder. Because the key phenotype of BD is a biphasic dysregulation in mood, behavior and sleep remain of great interest and could help expand the understanding of pathogenic mechanisms.

Sleep has a critical significance in the regulation of mood, and sleep disturbances can be seen in BD primarily or because of BD itself (7). These alterations have been linked to a lower quality of life, suicide attempts, poorer clinical and cognitive functioning, and higher relapse rates of mood episodes (8).

## Sleep Disorders and Bipolar Disorder: Epidemiology

The “sleep disorders” are defined as every significant alteration of quality of sleep, timing, and quantity, with different adverse impacts on function and quality of life (9). Sleep disturbances are very common in the general population (10). The prevalence of symptoms of sleep disorders range between 41% and 52% worldwide, with the most widespread symptoms being insomnia, followed by excessive daytime sleepiness, nightmares, difficulty falling asleep or maintaining sleep, poor sleep quality, sleep talking, sleep walking, and obstructive sleep apnea (11).

Sleep disorders also have a high prevalence in the psychiatric population. Furthermore, sleep disturbances exert a negative impact on the course and treatment of every psychiatric illness, and aberrant sleep represents a core symptom of BD. For example, 23% to 78% of patients with BD have reported symptoms of hypersomnia (10). The circadian rhythm hypothesis of BD postulates that variability of the circadian rhythms represents a critical step in BD evolution, whereas disturbances in circadian rhythms are considered a core element for the onset and progress of BD (12, 13). It is universally acknowledged that the increased risk of suicidal ideation and manic switch is linked to insomnia (14, 15).

Sleep disturbances are frequent in BD patients in different phases of illness, including the euthymic state (16) and remission (17). These sleep aberrations are represented not only by insomnia but also by sleep–wake rhythm disorders, especially delayed sleep–wake phase disorders (18–20) albeit the disturbance pattern can change with the specific mood phase. During the manic state, most patients (66–99%) experience a reduced need for sleep (21–23) and longer sleep onset latency (7), and *vice versa* sleep deprivation is well known as a trigger factor for manic episodes (24). Likewise, in the depressive state, insomnia (40–100%) and hypersomnia (23–78%) are commonly observed (25–27). A prevalence of 32.4% of circadian rhythm sleep–wake disorders (CRSWD) was found in a sample of 127 patients affected by BD type I or II, whereas younger onset age of BD and family history of suicide were associated with CRSWD in BD patients (28). Meta-analyses of trials conducted on remitted BD patients demonstrated prolonged total sleep time, increased awakenings after sleep onset, greater variability of sleep–wake variables, and reduced sleep efficiency (16, 29).

Overall, all kinds of sleep disorders and parasomnias are very common especially in youth patients with BD (30). Thus, compared to the general population, youth with BD exhibit lower sleep efficiency, longer slow wave sleep, and reduced REM sleep, features that could affect the genesis and prognosis of the disorder (7, 31). Sleep disturbances may also be used as predictors of the onset of BD in a subset of high-risk young subjects (32).

## Circadian Rhythms and Bipolar Disorder

Several types of rhythms rule the human body. Based on the approximate duration, these rhythms can be classified as circadian (about of 24 h), infradian (of longer duration), and ultradian (of shorter length). Temporal organization of physiological, cellular, organ, biochemical, and behavioral processes is controlled by circadian clocks (33).

**Figure 1:**
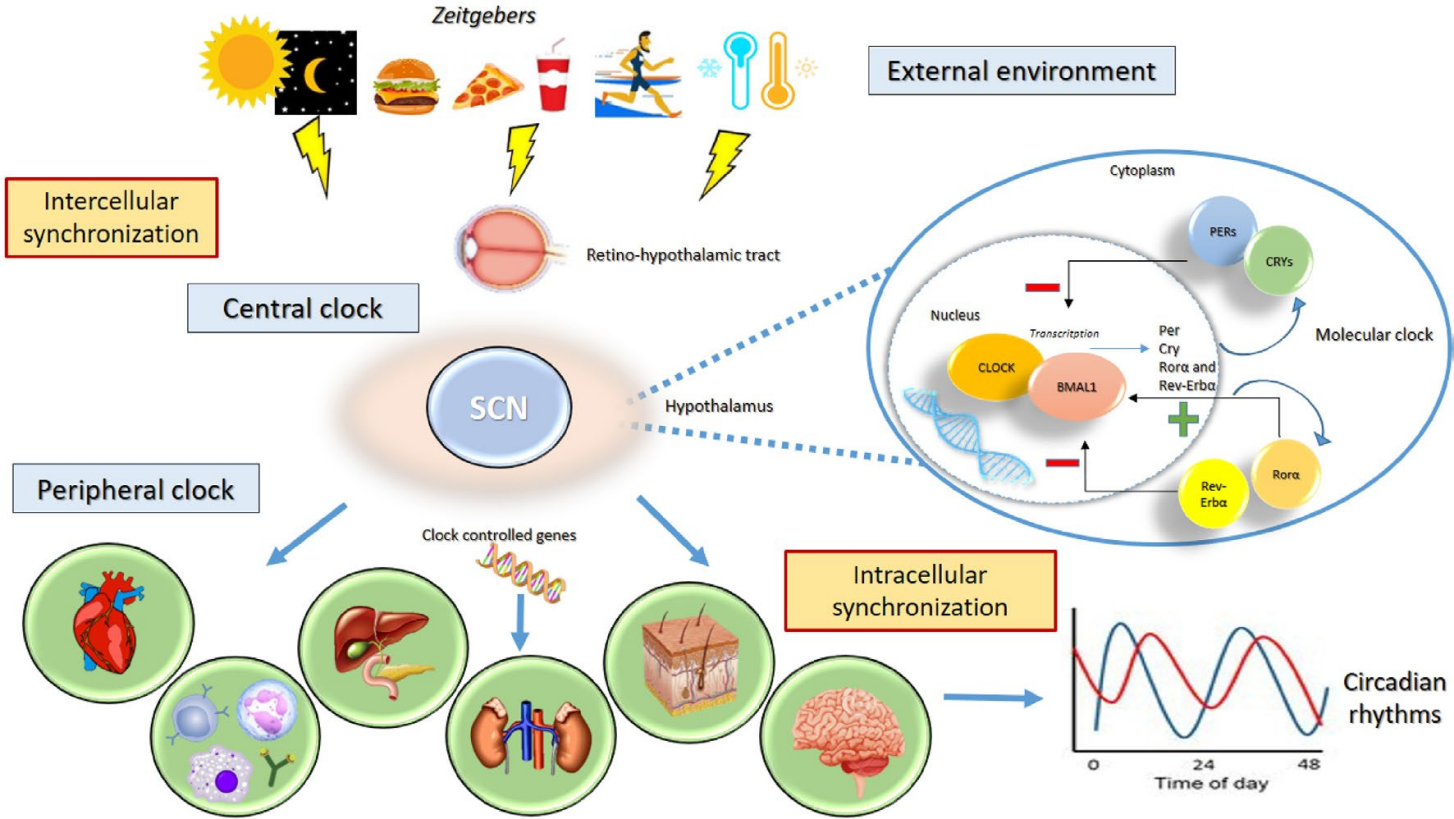
Circadian rhythms. The clock system captures exogenous “zeitgebers” (light/dark cycle, temperature, exercise, food intake) and triggers the central clock in the suprachiasmatic nucleus (SCN) of the hypothalamus through the retino-hypothalamic tract. The activity the group of clock genes governs the generation of circadian rhythms. The genes CLOCK and ARNTL encode the transcription factors CLOCK and ARNTL, which together activate the transcription of Per, Cry, RORα, and REV-ERBα genes. The proteins PER1, PER2, PER3, CRY 1, and CRY 2 combine to inhibit their own transcription, whereas RORα and REV-ERBα act on ARNTL to activate and inhibit transcription, respectively. The processed information is transmitted to the peripheral clocks and to other clocks in the brain to stabilize 24-h periodicity. The stable relationship between internal rhythms and the external environment is needed to ensure the synchronization of individual endogenous rhythms.

Endogenously generated circadian rhythms are tuned by and adapted to the environment so that the body is able to synchronize the internal time with the geophysical time. The clock system captures exogenous time signals, called “zeitgebers,” which include the day/night (or light/dark) cycle, temperature, and food intake (33). Environmental information is processed by a central clock, which is located in the anterior region of the hypothalamus, in the suprachiasmatic nuclei (SCN) (34). The central clock receives light and dark information from the visual input through the retino-hypothalamic tract; increased levels of light elevate alertness whereas decreased levels of light reduce sleep latency (35, 36). The processed information is transmitted to the peripheral clocks and to other clocks in the brain (located in other hypothalamic nuclei, thalamus, amygdala) to synchronize all individual endogenous rhythms (33, 37). The stable relationship between internal rhythms and the external environment is ensured by exposure to a normal light–dark schedule (**Figure 1**) (36). Lack of coordination between the endogenous circadian system and the sleep/wake cycle is a critical factor in the clinical status of illness associated to the disruption of the circadian timing of sleep and the alteration levels of alertness, vigilance, and performance (36, 38). In pathological conditions, the SCN and peripheral clocks lose their normal phase relationship, and thus, a state of internal desynchronization develops that, if sustained, may predispose individuals to a disease (36). The SCN received multiple feedbacks from the periphery that include information regarding metabolic status and the levels of activity (39).

Various pathological conditions are associated with sleep and circadian disturbances, including allergies, hypothyroidism and hyperthyroidism, coronary artery disease, congestive heart failure, diabetes, arthritis, asthma, gastroesophageal reflux disease, and chronic pain (40). Disorderly circadian system contributes to the etiology and progression of major psychiatric disorders (38, 41, 42). About three-quarters of individuals with delayed sleep phase syndrome have a past or current history of depression, whereas depression severity correlates with circadian misalignment (43). Patients with different psychiatric conditions, such as anxiety disorders and schizophrenia, often show circadian deregulation contributing to major functional impairments (44).

**Figure 2:**
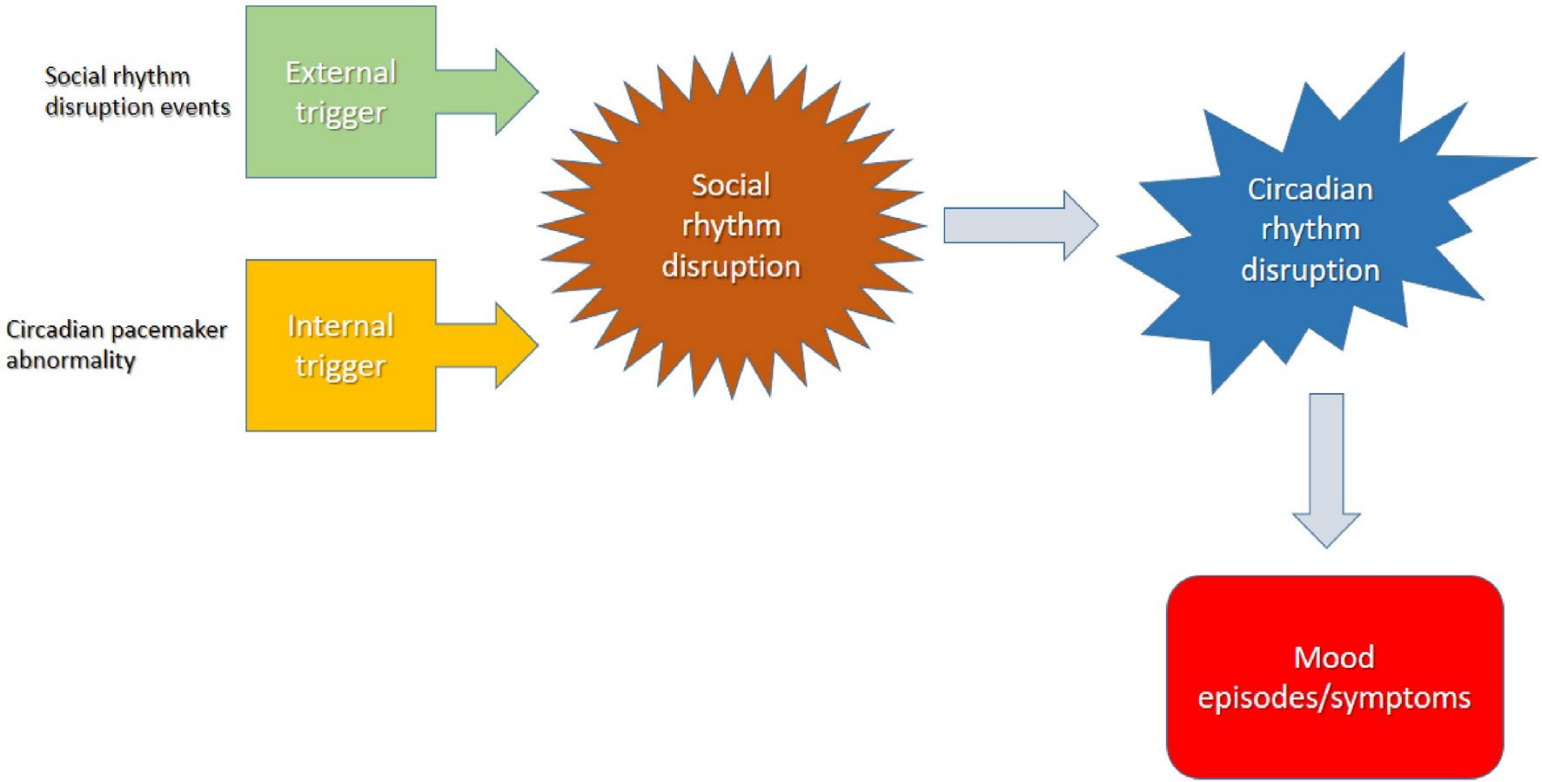
The disruption in sleep–awake cycle ends to precipitate or exacerbate mood episodes.

Sleep disturbances are common in BD with a great variability in sleep duration (45). The decreased need for sleep predicts the onset of a manic or hypomanic episode the following day (46), whereas sleep extension occurs frequently in the depressive episode (13, 16, 47, 48). The disruption in sleep–awake cycle tends to precipitate or exacerbate mood episodes (49), and they are risk factors for the recurrence of a mood episode (**Figure 2**) (50). Sleep deprivation was also found to induce manic episodes in animal BD models (51, 52). Loss of sleep confers a poor prognosis, increasing the risk of suicide in patients with a suicide attempt history (53). Even in euthymia, sleep alterations occur in BD patients (16). Given all this evidence and based on the rhythmic nature of BD, it has been suggested that the endogenous circadian system may play a role in BD etiology, clinical manifestations, and outcome (42, 54).

The activity of a group of clock genes governs the generation of circadian rhythms. There are molecular positive and negative transcriptional/translational feedback loops that drive the expression of different genes to stabilize 24-h periodicity (55). Several of these clock genes have been linked directly to the abnormal sleep/circadian phenotypes (36). Mutations of any of these circadian genes can potentially have an impact on the circadian clock and thus subtly or dramatically alter sleep, mood, or behavior in ways that contribute to physical and mental illness, and indeed many circadian genes have been associated with BD (56). The strongest evidence for genetic abnormalities is associated with polymorphisms of clock genes and an increased susceptibility to BD (57). In humans, genetic association studies of patients with BD have suggested that polymorphisms in the period gene (PER) is linked to specific phenotypes, such as a good lithium responder (36).

Particularly, a variant of PER3 gene has been linked to different chronotypes. The PER3 gene contains a variable number tandem repeat polymorphism, in which a 54-nucleotide coding-region segment is repeated four (PER^4/4^) or five (PER ^5/5^) times. The long allele variant of PER^5/5^ has been linked to extreme morning chronotypes, whereas the shorter allele PER^4/4^ is associated with extreme eveningness and delayed sleep phase syndrome (58). Incidentally, the early onset in BD is associated with the longer allele (PER^5/5^), whereas the later onset is associated with the shorter allele (PER^4/4^) (36) (**Table 1**).

**Table 1:** Main polymorphism of clock genes and their association in bipolar disorder.

Clock gene	Polymorphism	Association found in bipolar disorder	References
PER3	rs57875989	Extreme morning and early onset	Dijk and Archer (58)
		Extreme eveningness and later onset	
PER 2	rs934945	Diurnal preference for eveningness	Song et al. (59)
CLOCK	rs10462028	Association with BD aetiology	Nievergelt et al. (60) Dmitrzak-Weglarz et al. (61) Soria et al. (62)
	rs11932595	Sleep disorders	Maciukiewicz et al. (63)
	rs1801260	Early stress, history of suicide attempt and persistence of the idea of suicide	Benedetti et al. (64) Bollettini et al. (65)
	rs11932595	More depressive episode and appetite disorder	Maciukiewicz et al. (63)
	rs1801260	Influence on sleep pattern, daytime preference, age at onset, and response to treatment	Bollettini et al. (65)
ARNTL (BMAL1)	rs2279287 rs1481892 rs1982350	Seasonal pattern Higher susceptibility to the disease	Geoffroy et al. (66) Rajendran and Janakarajan (67)
TIMELESS	rs2291738 rs10876890	Suicide attempts Insomnia	Pawlak et al. (68)
PPIEL*	Lower methylation level	Altered dopaminergic transmission or neuroendocrine system functions	Kuratomi et al. (69)
NR1D1 promoter	rs2071427	Good response to treatment	McCarthy et al. (70)
CRY1	rs8192440	Good response to treatment	McCarthy et al. (70)
GSK-3β	rs6438552	Robust and additive response to treatment if associated with NR1D1 (rs2071427)	Oliveira et al. (71)

ARNTL (BMAL1), Aryl hydrocarbon receptor nuclear translocator like protein-1; CRY 1-2, Cryptochrome circadian regulator; GSK-3β, Glycogen synthase kinase-3; NR1D1, Nuclear receptor subfamily 1 group D member 1; PER3, Period circadian regulator-3; PPIEL, E-like peptidilprolil isomerase; TIMELESS, Timeless Circadian Clock. *pseudogene.

One of the consequences of sleep/circadian disruption is an abnormality in the stress axis, with particular emphasis on atypical neurotransmitter release. The hypercortisolemia can arise from a breakdown in glucocorticoid receptor-mediated negative feedback mechanisms in the hypothalamic–pituitary–adrenal (HPA) axis (36). Circadian disturbances, such as a phase advance of the diurnal rhythm of plasma melatonin (72) and plasma cortisol (73), have been observed in BD, although these were not universally confirmed (74). In relation to oxidative stress, circadian rhythm disturbance was associated with increased lipid peroxidation in BD (75). Studying alteration of the wake–sleep rhythm may provide yet unknown insights into the pathophysiology of BD.

## Neuroglia in Bipolar Disorder and Sleep Disorders

### Neuroglia: An Overview

Neuroglia represent the homeostatic and defensive arm of the nervous system; neuroglial cells of the central nervous system (CNS) are classified into astrocytes, microglia, and oligodendrocytes and their precursors, also known as NG2 glia (76). The functions of neuroglia are diverse; these nonexcitable cells are indispensable companions of neurons, supporting them in physiology and protecting them against pathological lesions. Astrocytes are the main homeostatic cells of the CNS, which control the homeostasis of the nerve tissue at all level of organization from molecular to organ (77, 78). Astroglial perisynaptic processes cover synaptic contacts and form synaptic cradle, which through various mechanisms control synaptogenesis, synaptic maturation, synaptic maintenance, and synaptic extinction (79). Microglial cells invade the neural tube early in development and are fundamental for early shaping of neuronal connections by synaptic stripping (80). Finally, oligodendrocytes support and protect axons and provide for gray and white matter myelination, which supports brain connectome (81). The fundamental role of neuroglia in neuropathology has been considered by many prominent neuroanatomists (including Santiago Ramon y Cajal, Alois Alzheimer, Nicolas Achucarro, and Franz Nissl, to name a few) a century ago. The recent decade has witnessed the revival of interest to pathological potential of neuroglia, challenging universally accepted neurono-centric neuropathological doctrine (82–86).

### Pathological Classifications of Neuroglia

Conceptually, neuroglial cells contribute to all neurological diseases either as primary elements driving pathology or by responding to lesions through an evolutionary conserved defensive program of reactive gliosis. Neuroglial changes in pathological conditions are context- and disease-specific, are complex, and evolve through the stages of neuropathology. Astrogliopathology in particular is subclassified (86) into i) reactive astrogliosis, which represents a graded response to various types of lesions.Reactive astrogliosis is fundamentally neuroprotective and produces a wide spectrum of reactive phenotypes that are disease- and disease stage-specific (84, 86–90); ii) pathological remodeling of astrocytes—when astrocytes acquire new properties driving neuropathology, Alexander disease (91) being a signal example; and iii) astroglial atrophy and loss of function. Similarly, microglial cells in pathology assume a multitude of phenotypes with various degrees of activation with both neuroprotective and neurotoxic functions. In chronic pathologies, microglial cells often undergo degeneration that limits their defensive capabilities (92, 93) or pathological remodeling (94). Pathological classification of oligodendrocytes is yet to be produced.

### Neuroglial Abnormalities in Psychiatric Disorders

Neuroglial abnormalities are widely manifested in all major psychiatric diseases; and they are particularly prominent in bipolar disease and in major depression (95–98). In contrast to many other neuropathologies, there are no signs of astroglial reactivity in BD (as well as in other major psychiatric diseases); instead, astrocytes demonstrate prominent atrophy and asthenia, which most likely is associated with loss of homeostatic and supportive functions that in turn underlie failures in information processing and neurotransmission. Already in early stereological studies using Nissl staining (that revealed a total glial population), a prominent decrease in the overall number of neuroglial cells has been described in human postmortem samples from both major depressive disorder and BD (99). Subsequent morphometric studies have confirmed a significant reduction in glial numbers (up to 20–40%) in relevant brain regions (including the prefrontal cortex, orbitofrontal cortex, subgenual cortex, anterior cingulate cortex, and amygdala) in BD and major depression (95, 100–104). The expression of glial fibrillary acidic protein (GFAP), the marker of astroglial reactivity, which reveals the cytoskeleton of astrocytes, is generally suppressed in brain samples from young or adult subjects with depression and BD (105–107). In older subjects, GFAP expression was sometimes increased, which reflects general age-dependent changes or neuroinflammatory changes (105). Very significant (up to 95%) decrease in GFAP expression and GFAP-positive astroglial profiles have been recently detected in the white matter of the ventral prefrontal cortex of subjects with major depression (108). Impairment of astroglial networks and aberrant signaling in astroglial syncytia were evidenced by a significant decrease in the expression of major astroglial connexins XC30 and Cx 43 in the prefrontal cortex of depression-associated suicide victims (109). Major depression (but not BD) was found to be associated with a significant decrease in the density of astrocytes expressing glutamine syntethase and with downregulation of astroglial expression of glutamate transporter GLT-1, suggesting thus aberrant operation of glutamine–glutamate shuttle (110, 111). Likewise, the population of S100B-positive astrocytes was decreased in hippocampi of patients with BD and major depression (112).

Similar reduction in glial numbers and GFAP expression and astroglial morphological profiles have been detected in animal models of depressive behavior. These models are often based on exposure of animals to various types of chronic stress that instigate depressive-like behavior manifested by anhedonia or aberrant social communications. The density of GFAP-positive astrocytes and morphological astroglial profiles were reduced after the stress of separating juveniles from their family (113), chronic social defeat (114), or chronic mild stress (115), which induces prominent morphological atrophy of astroglial cells (116). Astroglial atrophy in chronic stress animal models may be associated with aberrant glycogen processing and decreased glycogen content (117). Significant astroglial atrophy was also observed in the repeated corticosterone injection-induced mouse depression model (118). Likewise, the density of astrocytes was significantly reduced in the prefrontal cortex, anterior cingulate cortex, amygdala, and hippocampus of Wistar–Kyoto strain of rats susceptible to depressive-like behavior (119). Chronic stress-induced astroglial asthenia and loss of function, as well as depressive behavior, were reversed by treating animals with riluzole, the drug that limits glutamate excitotoxicity (115). Selective ablation of astrocytes after injection of L-α aminoadipic acid into either rodent prefrontal cortex or prelimbic cortex triggered depressive-like behavior (120, 121); injection of neuronal toxin ibotenate had no such an effect (120). Emergence of depressive phenotype is associated with astroglia-specific decrease of expression of several genes associated with signaling systems, including serotonin 5-HT_2B_ receptors, cytosolic phospholipase 2α, ionotropic kainate receptor GluK2, and adenosine deaminase acting on RNA 2 (ADAR2); treatment with fluoxetine restored altered expression (122, 123). The chronic stress-induced depressive phenotypes were also linked to a downregulation of astroglial expression of multiple endocrine neoplasia type 1 gene encoding protein menin; the efficiency in menin was associated with increased activation of NF-κB activation and elevated production of IL-1β (124). Depression after traumatic brain injury was associated with a decrease in astroglial expression of glutamate transporters (125), this being another example of astroglial asthenia with loss of function. All in all, these data underlie the hypothesis of the role of astroglial asthenia in the pathophysiology of mood disorders, including BD (97, 126, 127).

**Figure 3:**
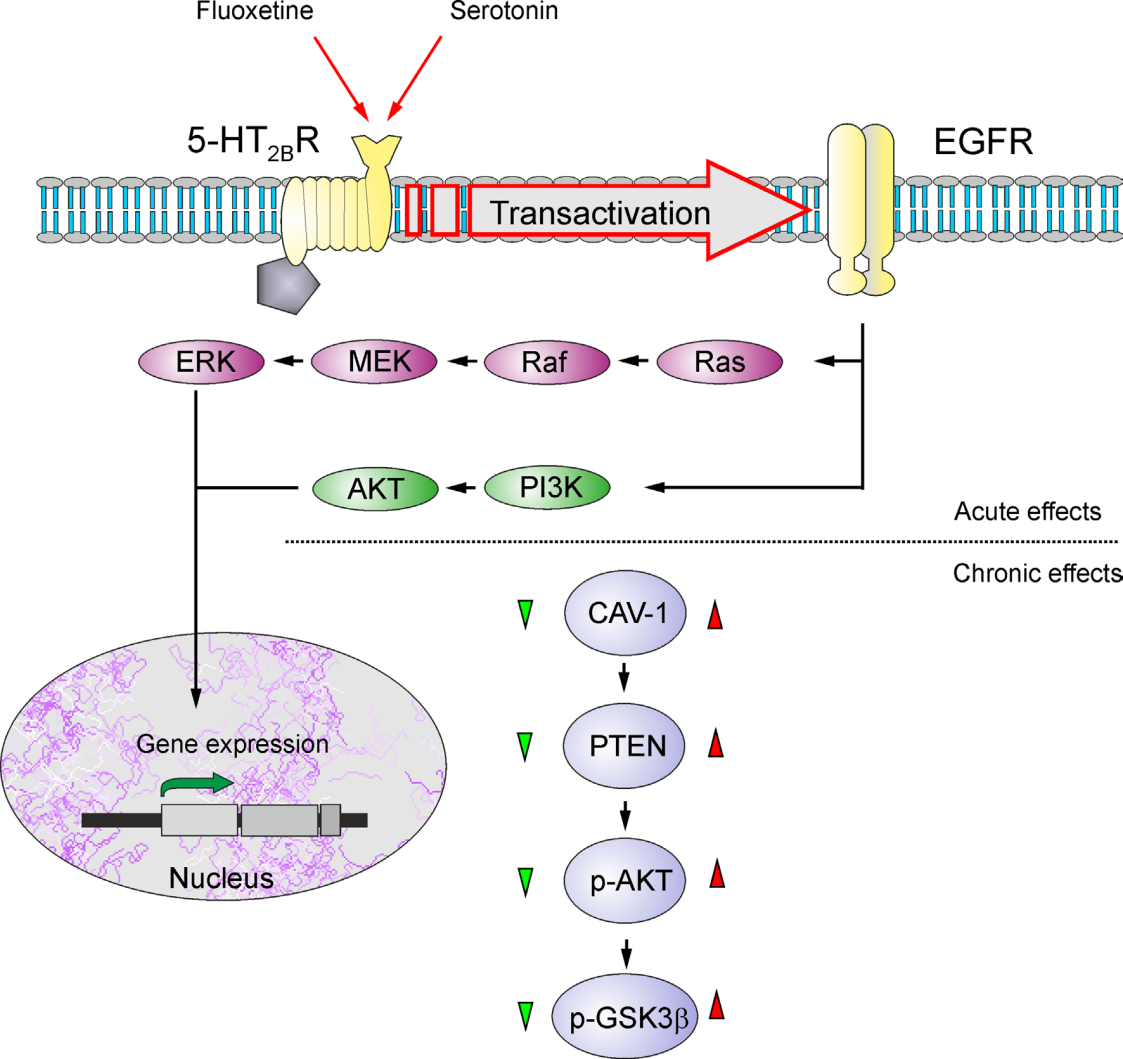
Schematic illustration of biphasic concentration-dependent regulation of Cav-1 gene expression and GSK-3β activity by fluoxetine in astrocytes. Acute treatment with fluoxetine stimulates 5-HT_2B_ receptors, induces transactivation of EGF receptors and activates its MAPK/ERK and PI3K/AKT signal pathways that, in turn, regulate gene expression in astrocytes. Chronic treatment with fluoxetine at low concentrations (green triangle) decreases Cav-1 gene expression, which decreases membrane content of PTEN, induces dephosphorylation and inhibition of PI3K and elevates GSK-3β phosphorylation thus suppressing its activity. At higher concentrations (red triangle) fluoxetine increases Cav-1 gene expression that acts on PTEN/PI3K/AKT/GSK-3β in an inverse fashion. Reproduced from Ref. (139).

Astrocytes are recognized as therapeutic targets for the treatment of psychiatric disorders and, in particular, depression and BD (128–130). Treatment of animals subjected to psychosocial stress prevented the loss of astrocytes (114), whereas riluzole (the drug that limits glutamate excitotoxicity) similarly prevented loss of astrocytes in animals subjected to mild chronic stress (115). Even electroconvulsive therapy (ECT) has been shown to increase the expression of GFAP in the piriform cortex, amygdala, and hippocampus (131). Recent findings identified astrocytes as primary targets for transcranial direct current stimulation used for the management of depression (132). Moreover, it has been documented that two classical mood stabilizers used as first-line therapy for BD, lithium (Li^+^) and valproic acid (VPA), have a neuroprotective role reducing neuroinflammation through modulating the activation of astrocytes (133). Chronic treatments of astrocytes *in vitro* with Li^+^, VPA, and another classic antidepressant, carbamazepine (CBZ), suppress glutamate release, thus contributing to alleviation of excitotoxicity (134). Long-lasting exposure of astrocytes to antidepressant fluoxetine, a selective serotonin reuptake inhibitor, increased cytosolic pH from 7.18 to 7.58 by stimulating sodium-proton transporter 1, thus affecting brain pH homeostasis (135). Fluoxetine, as well as Li^+^, VPA, and CBZ, also affects astroglial glycogen content in a concentration-dependent manner, increasing glycogen at low concentrations and decreasing at high concentrations—this action being mediated by caveoline-1 (Cav-1) - phosphatase and tensin homologue (PTEN) - phosphoinositide 3-kinase (PI3K) - glycogen synthase kinase 3 (GSK-3β) cascade (**Figure 3**) (136, 137). These multiple actions of fluoxetine on astrocytes are mediated through direct activation of serotonin 5-HT_2B_ receptors and transactivation of epidermal growth factor receptor (EGFR) (138, 139). Chronic treatment with antidepressants, as well as stimulation of adrenoceptors, was also reported to stimulate astroglial secretion of brain-derived neurotrophic factor (BDNF), which may boost synaptic transmission and provide neuroprotection (140, 141).

Analysis of lipopolysaccharide (LPS)-induced inflammation in rat primary mixed (80% astrocytes and 15% microglia) glial cultures found that Li^+^ decreases the secretion of TNF-α, IL1-β, prostaglandin E2, and nitric oxide (142). Pretreatment of LPS-stimulated microglial cells with Li^+^ significantly inhibited LPS-induced microglial activation and proinflammatory cytokine production (143). Similarly, VPA modulates microglial response to inflammatory insults mediated by LPS and may affect the synaptic excitatory inhibitory balance through its effect on astrocytes in rats (144, 145).

## Astrocytes and Sleep Regulation

The role of astroglia in the regulation of sleep has been suggested more than a century ago by Santiago Ramon y Cajal, who suggested that astroglial processes, by entering the synaptic cleft, may slow down communication in neuronal networks, thus instigating sleep (146); a very similar mechanism was also considered by Carl-Ludwig Schleich (147) as a basis for general anesthesia.

Astrocytes of the suprachiasmatic nucleus do contain clock genes and do produce circadian rhythms of GFAP expression; astrocytes, in addition, may contribute to timekeeping through regulating glutamate levels (148). Nonetheless, it seems that the major role of astrocytes is the regulation of sleep homeostasis. The latter refers to a regulation mechanism that increases urge to sleep proportionally to the time spent awake (149). Sleep homeostasis is regulated by accumulation of adenosine in the brain during wakefulness (150), and the data accumulated demonstrated that the main source for adenosine in the physiological conditions is associated with astrocytes (151). Another important role of astroglia in sleep is associated with cleansing the brain parenchyma (152). It is, therefore, plausible to speculate that astroglial asthenia observed in mood disorders and in BD impairs astroglial sleep-regulating capabilities.

## Sleep, Astroglia, and Bipolar Disorder

As has been mentioned above, sleep plays a key role in the clinical manifestations of BD. Alterations in the structure or duration of sleep are reported in all phases of the disorder—in the manic, depressive, and euthymic phases (65). During manic or hypomanic episodes, there is a reduced need for sleep, whereas during depressive episodes, there may be difficulty in achieving adequate quality or amount of sleep or, alternatively, patients experience hypersomnia (12, 153). Sleep abnormalities are strongly associated with immune dysfunction. Aberrant sleep is associated with increased levels of proinflammatory cytokines with a bidirectional causal association identified (154, 155). As such, interest has grown in immune dysfunction as a potential link that underwent two-way interaction between sleep dysfunction and BD (156, 157). Both postmortem and *in vivo* studies showed that microglial activation is involved in the neurobiology of BD (158, 159). These findings agree with the presence of peripheral inflammatory markers and the blood–brain barrier disruption revealed by meta-analyses. If as it seems it is true that modifications of inflammatory markers and microglial function may play an important role in progression of BD, several drugs used in the treatment of this disorder could have effects on glial cells, and future studies may use these cells as targets for the development of new treatments in this way (160, 161).

## Conclusion

Sleep disturbances are common in patients with BD; these sleep alterations are present even during euthymia, as insomnia, increased sleep latency, and variability in sleep hours. Recent research has sought to identify the biological markers that underlie sleep disorders in patients with BD. The focus of the latest studies has highlighted the role for neuroglial cells. Astrocytes, the primary homeostatic cells of the CNS, undergo atrophy, asthenia, and loss in BD-specific brain regions, and deficiency in glial support and neuroprotection may have a key role to the pathophysiology of BD (84, 160), even though the precise mechanisms need to be further explored and clarified. Several drugs used for the treatment of BD have specific effects on glial cells indicating neuroglia as a target for the development of new treatments. Further research should concentrate on investigations of glial cells *in vivo* and in “humanized” preparations to increase our understanding of the role of glia in sleep regulation in people with BD. Additional systematic studies are also needed to highlight the importance of sleep disorders in patients with BD to offer a tailor-made treatment for these patients.

## Author Contributions

LS, AV conceived the manuscript. LS, AV, RF, EC wrote the manuscript. LS, AV, CS-G, PDF edited the text and supervised the paper. The manuscript was critically revised and finally approved by AV, LS, CS-G and PDF. LS and AV coordinated the work.

## Conflict of Interest Statement

The authors declare that the research was conducted in the absence of any commercial or financial relationships that could be construed as a potential conflict of interest.
